# Bergenin, a bioactive flavonoid: advancements in the prospects of anticancer mechanism, pharmacokinetics and nanoformulations

**DOI:** 10.3389/fphar.2024.1481587

**Published:** 2025-01-06

**Authors:** Pratibha Pandey, Sorabh Lakhanpal, Danish Mahmood, Han Na Kang, Byunggyu Kim, Sojin Kang, Jinwon Choi, Seungjoon Moon, Shivam Pandey, Suhas Ballal, Sanjay Kumar, Fahad Khan, Bonglee Kim

**Affiliations:** ^1^ Centre for Research Impact and Outcome, Chitkara University Institute of Engineering and Technology, Chitkara University, Rajpura, India; ^2^ Chitkara Centre for Research and Development, Chitkara University Himanchal Pradesh, Baddi, India; ^3^ School of Pharmaceutical Sciences, Lovely Professional University, Phagwara, India; ^4^ Department of Pharmacology and Toxicology, College of Pharmacy, Qassim University, Buraydah, Saudi Arabia; ^5^ KM Convergence Research Division, Korea Institute of Oriental Medicine, Daejeon, Republic of Korea; ^6^ Department of Pathology, College of Korean Medicine, Kyung Hee University, Seoul, Republic of Korea; ^7^ School of Applied and Life Sciences, Uttaranchal University, Dehradun, India; ^8^ Department of Chemistry and Biochemistry, School of Sciences, JAIN (Deemed to be University), Bangalore, India; ^9^ Department of Allied Healthcare and Sciences, Vivekananda Global University, Rajasthan, India; ^10^ Center for Global Health Research Saveetha Medical College, Saveetha Institute of Medical and Technical Sciences, Chennai, India

**Keywords:** bergenin, anticancer, pharmacokinetic, cancer, therapeutics

## Abstract

The natural world is a vast reservoir of exceptionally varied and inventive chemical compositions. Natural products are used as initial compounds to create combinatorial libraries by targeted modifications and then by analyzing their structure-activity connections. This stage is regarded as a crucial milestone in drug discovery and development. Bergenin, a naturally occurring secondary metabolite, has been extracted from several plant components. It is a constituent found in herbal and Ayurvedic preparations. It demonstrates antiviral, antifungal, antitussive, antiplasmodial, anti-inflammatory, antihepatotoxic, antiarrhythmic, antitumor, antiulcerogenic, antidiabetic, and wound healing activities. Bergenin efficiently inhibited the proliferation of human cancer cells by stimulating the production of intracellular reactive oxygen species (ROS), causing DNA damage and leading to cell cycle arrest in the G1/G2 phases by blocking cell signaling pathways. More comprehensive reviews are needed on the anticancer properties of bergenin. Therefore, our review aimed to update the multifaceted benefits of bergenin to the future scientists and researchers, which can be leveraged to formulate safer and novel cancer therapies, while also establishing a robust framework for future investigations into bergenin in cancer treatment. More preclinical and clinical investigations are needed to validate the candidature of bergenin as a potent anticancer agent.

## 1 Introduction

**FIGURE 1:**
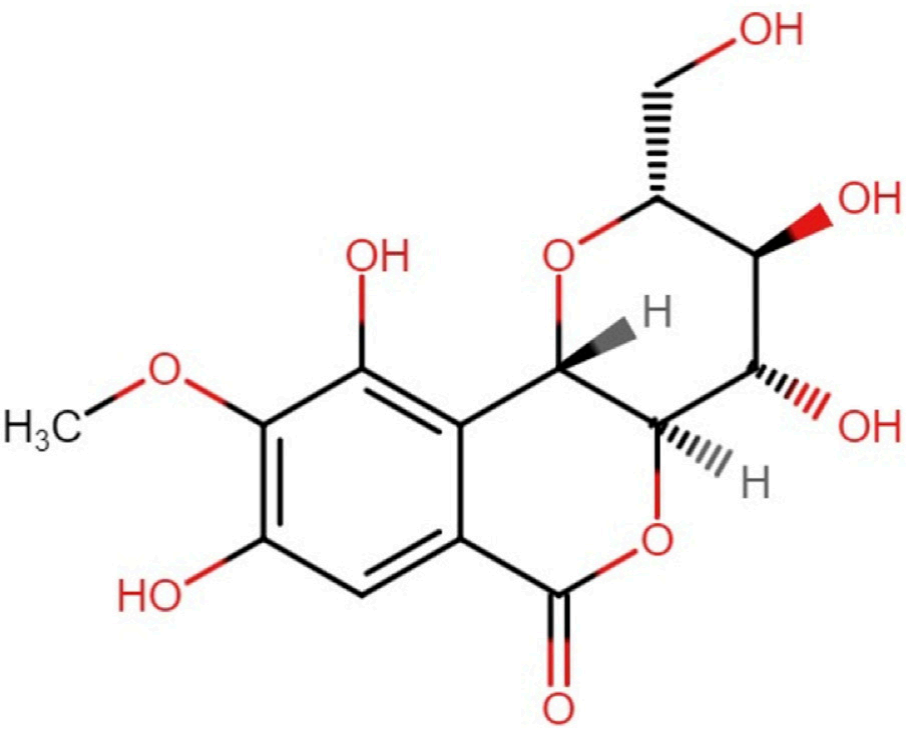
Chemical structure of Bergenin [C_14_H_16_O_9_].

Bergenin is a bioactive compound found in plants belonging to the genus Bergenia. Studies have shown that bergenin possesses several biological actions, such as anti-inflammatory and immunomodulatory characteristics. Bergenin offers a new and innovative option as an antioxidant, with potential uses in multiple sectors such as pharmaceuticals, cosmetics, and food production (Koul et al., 2020). Bergenin is a compound that consists of trihydroxybenzoic acid and is a derivative of 4-O-methyl gallic acid. It functions as a metabolite. This compound is a hydrolysable phenolic glycoside that is synthesized as colorless crystals. It has limited solubility in water and readily degrades in essential solutions and its stability relies on the circumstances in which it is stored (Salimo et al., 2023). Tschitschibabin et al. (1929) were the first to propose the structure of bergenin (Tschitschibabin et al., 1929). Initially, a chemical structure consisting of merely two rings and a single aliphatic chain was suggested (Ye et al., 2004). Subsequently, in 1950, Shimokoriyama made revisions to the structure and put out a proposal consisting of three rings, each composed of six members. Bergenin possesses five hydroxyl groups deemed to have potential activity (Figure 1). Takahashi et al. synthesized a range of Bergenin derivatives with significantly improved antioxidant activity by chemically catalyzing the esterification of hydroxyl groups using various fatty acids (Takahashi et al., 2003).

It is extracted from many plants, including Bergenia crassifolia (B. crassifolia), Corylopsis spicata (C. spicata), Caesalpinia digyna (C. digyna), Mallotus japonicus (M. japonicas), Sacoglottis gabonensis (S. gabonensis), and among others (Salimo et al., 2023) (Table 1). Several researchers have examined various elements of this plant-derived molecule, but no comprehensive review provides specific information on the medicinal potential of bergenin (Bajracharya, 2015). This review represents the initial effort to provide information on the efficacy of this remarkable herb in combating various types of human carcinomas. However, this method is constrained by the insufficient and inconsistent levels of bergenin found in these medicinal plants. Furthermore, the chemical synthesis of bergenin encounters numerous obstacles, particularly regarding the selective placement of C-sugar and O-methyl groups in certain regions or with specific stereochemistry.

**TABLE 1:** Various plant sources of bergenin.

Plant	Family	Yield (%)	Reference
*Ardisia japonica*	Myrsinaceae	0.0225	Li et al. (2005)
*Caesalpinia digyna*	Caesalpiniaceae	0.0650	Roy et al. (2012)
*Bergenia ciliate*	Saxifragaceae	0.0760	Kashima & Miyazawa. (2012)
*Shorea robusta*	Dipterocarpaceae	0.0750	Mukherjee et al. (2013)
*Bergenia cordifolia*	Saxifragaceae	0.5688	Roselli et al. (2012)
*Endopleura uchi*	Humiriaceae	1.1347	Borges et al. (2011)
*Mallotus japonicas*	Euphorbiaceae	1.9500	Zhang et al. (2003)
*Garcinia malaccensis*	Guttiferae	0.0012	Alkadi et al. (2013)
*Securinega virosa*	Euphorbaiceae	0.0122	Sanogo et al. (2009)
*Rodgersia sambucifolia*	Saxifragaceae	3.1250	Deng et al. (2010)
*Diospyros sanja-minika*	Ebenaceae	3.6402	Musgrave and Skoyles (1974)
*Peltophorum pterocarpum*	Fabaceae	0.1008	Raj et al. (2012)
*Brachystemma calycinum*	Caryophyllaceae	0.0002	Zhao et al. (2011)
*Bergenia stracheyi*	Saxifragaceae	2.0425	Nazir et al. (2011)

## 2 Biosynthesis of bergenin

**FIGURE 2:**
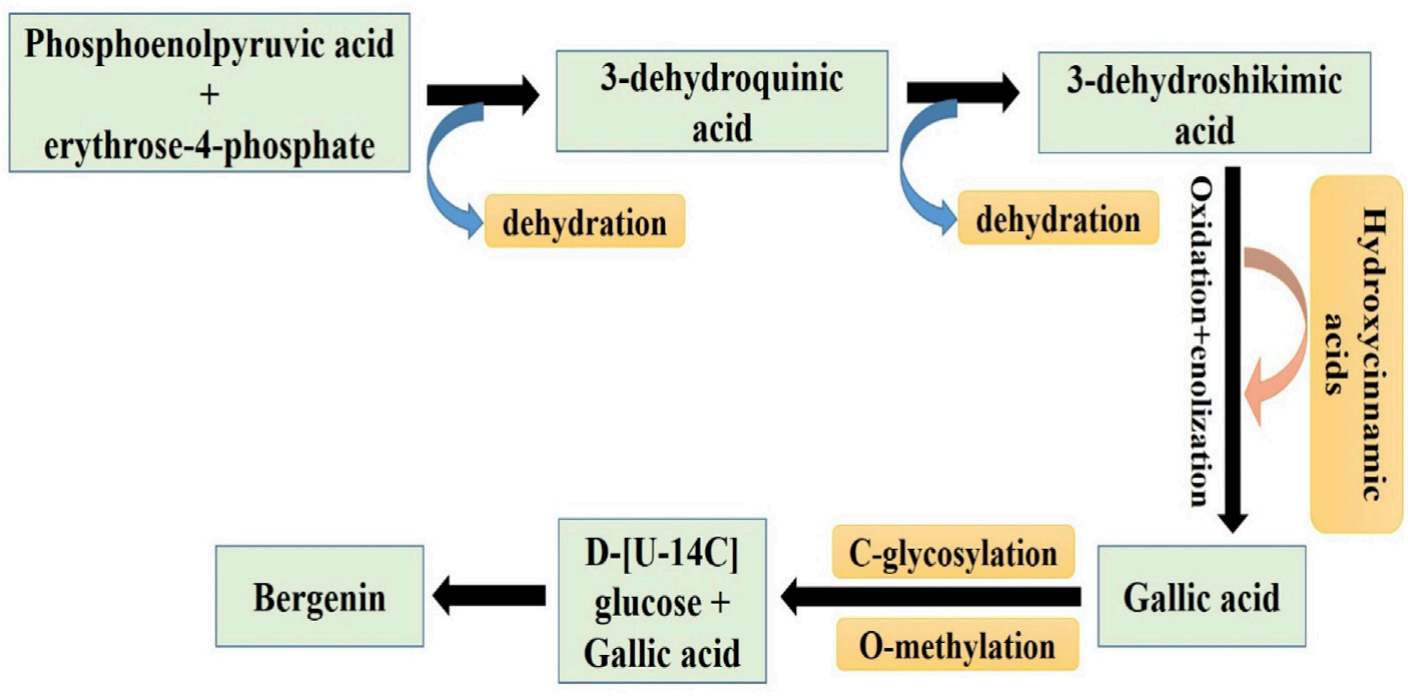
Pictorial representation of the Sequential pathways involved in the synthesis of Bergenin from phosphoenolpyruvic acid and erythrose-4-phosphate (related to gallic acid biosynthetic pathway) (Patel et al., 2012).

The synthesis of bergenin is intricately linked to the synthesis of gallic acid, which arises from the fusion of erythrose-4-phosphate with phosphoenolpyruvate, resulting in the production of 3-dehydroquinic acid. The dehydration of the latter compound resulted in the production of 3-dehydroshikimic acid, which gets further transformed into gallic acid by oxidation and enolization processes (Patel et al., 2012). Additional potential pathways included the breakdown of the side chain of hydroxycinnamic acids or the combination of an acetyl-CoA group with three malonyl-CoA units. Previous experiments have yielded conflicting results about the C-glycosylation phase. Isotopically labeled benzoic acid and hydroxycinnamic acids have indicated that C-glycosylation is more likely to occur in C6-C3 compounds rather than in C6-C1 derivatives like benzoic acid. Alternatively, when D-[U-14C] glucose is combined with labeled gallic acid, it demonstrates that the C-glycosylation process primarily occurs in the C6-C1 derivative (Taneyama and Yoshida, 1979). The glycosidic component undergoes condensation with the carboxylic acid of the gallic acid section, producing the lactone portion seen in the bergenin structure. Recent investigations indicate that the C-glycosylation process precedes the O-methylation of the phenolic hydroxyl of the gallic acid-derived part by O-methyltransferases (Yan et al., 2024) (Figure 2).

Various bergenin derivatives have been extracted from plants, although not all exhibited significant biological activities (Nazir et al., 2011). The primary compounds obtained from bergenin that have this particular potential are demethylated analogues or those that are esterified with phenolic acids such as gallic acid. Notable natural derivatives of bergenin include “riverbergenin” (Ali et al., 2011). Norbergenin was extracted from the leaves of *A. japonica* and separated from the trunk of *R. hypocrateriformis*. 11-O-galloylbergenin was isolated from the rhizome of *A. gigantifolia*, 11-O-veratroylbergenin was extracted from the rhizome of *A. gigantifolia*, whereas 4-O-galloylnorbergenin was obtained from the stem bark of *M. japonicus*. 8-O-methylnorbegenin is derived from all parts of *S. stolonifera*, 11-O-acetylbergenin is obtained from the aerial parts of *F. virosa*, and 11-O-vanilloylbergenin is extracted from the roots of A. crenata. Three compounds, namely, 4-O-syringoylnorbergenin, 11-O-p-hydroxybenzolynorbergenin, and 4-O-(3′-O-methylgalloyl) norbergenin were isolated from the stem bark of *D. sanza-minika* (Costa et al., 2021). Furthermore, certain semisynthetic derivatives have been specifically developed to enhance their pharmacological capabilities, necessitating the proposal of various alterations.

## 3 Pharmacokinetics of bergenin

Pharmacokinetic studies are essential for determining the effectiveness of natural medications and can also reveal the active ingredients. The pharmacokinetic investigation conducted in the past 10 years has confirmed the intracorporal mechanisms of bergenin (Table 2).

**TABLE 2:** Physicochemical and pharmacokinetic Properties [Values are obtained from SWISS ADME].

Parameters	Values
PubChem ID	12105
Volume	293.597
Molecular weight	328.27 g/mol
Density	1.117
Bioavailability score	0.55
Solubility	Water soluble
H-Bond Acceptor	9
H-Bond Donor	5
Rotatable bonds	2
GI absorption	Low
CYP2C19 inhibitor	No
GI absorption	Low
P-gp Substrate	No
CYP2C9 inhibitor	No
BBB permeant	No
Log Kp (skin permeation)	−8.99 cm/s
CYP1A2 inhibitor	No
CYP3A4 inhibitor	No
Lipinski	Yes
Synthetic accessibility	4.39

Following data was obtained using smile (OC [C@H]1O [C@@H]2 [C@@H]([C@H]([C@@H]1O)O)OC(=O)c1c2c(O)c (c (c1)O)OC) (From PUBCHEM database) in the SWISS ADME server (http://www.swissadme.ch/index.php) to analyses the pharmacokinetics, drug likeness, and physicochemical properties needed to validate its candidature for drug designing. In Table 2, several parameters including molecular weight, H bond donor and acceptor is mentioned to validate the drug likeness potential of bergenin. Furthermore, scientists also carried out a pharmacokinetic investigation of bergenin when delivered orally to rats at 22.5 mg/kg as a single dose. The findings suggested that bergenin underwent rapid degradation in the digestive system, including a first-pass metabolism, and exhibited limited absorption from the gastrointestinal tract in rats. The absorption parameter of any substance can be evaluated using Caco-2 permeability, P-glycoprotein inhibitors, P-glycoprotein substrates, and human intestinal absorption (HIA). Prior to entering systemic circulation, an oral medication must traverse intestinal cell membranes through passive diffusion, carrier-mediated absorption, or active transport mechanisms. The human colon adenocarcinoma cell lines (Caco-2) serve as a viable model for the human intestinal epithelium and are frequently employed to assess *in vivo* drug permeability owing to their morphological and functional resemblances. Consequently, Caco-2 cell permeability has emerged as a significant criterion for a viable candidate therapeutic molecule. The anticipated Caco-2 permeability of bergenin is −6.191 log cm/s, and it is measured at < −5.15 log cm/s, indicating that bergenin exhibits poor Caco-2 permeability. The P-glycoprotein inhibitor is a membrane protein that belongs to the ATP-binding cassette transporter superfamily. Bergenin is a highly effective inhibitor of P-glycoprotein. The modulation of P-glycoprotein-mediated transport has considerable pharmacokinetic consequences for P-glycoprotein substrates, which may be utilized for particular therapeutic benefits or lead to contraindications. Bergenin possesses the highest likelihood of being employed as a P-glycoprotein substrate. The absorption of an oral medicine in the human intestine is a fundamental requirement for its perceived efficacy. HIA serves as a potential alternative measure for oral bioavailability, and it is evident that bergenin exhibits inadequate human intestine absorption (less than 30%). A key route of drug absorption and distribution is plasma protein binding (PPB), hence, the affinity of a medication for plasma proteins significantly affects its pharmacodynamic properties. PPB can directly affect oral bioavailability as the free concentration of the drug is compromised when it binds to serum proteins during this process. Bergeninn is stated to possess a proper PPB of 33.498% (predicted value <90%) and has a low therapeutic index. Pharmaceuticals that exert effects on the central nervous system must traverse the blood-brain barrier to access their molecular targets. Conversely, for medications targeting peripheral sites, minimal or no blood-brain barrier penetration may be necessary to prevent central nervous system side effects. Bergenin possesses a modest blood-brain barrier (BBB) permeability, hence restricting its efficacy for central nervous system (CNS) illnesses. Clearance is a crucial pharmacokinetic parameter that, along with the volume of distribution and half-life, determines the dosage frequency of a drug. The clearance value of the bergenin compound is reported to be 8.395 mL/min/kg, indicating a high clearance rate. The half-life of a medication is a composite notion that encompasses clearance and volume of distribution; thus, obtaining accurate estimates of these two qualities is probably more pertinent. The Bergenin compound has a half-life of 0.927.

Following oral ingestion in humans, bergenin was rapidly but partially absorbed, exhibiting a brief half-life and limited bioavailability. According to a study conducted by Jiangsu New College Med, the maximum concentration (Cmax) was observed in plasma 1–4 h after intramuscular dose in dogs. In urine, the Cmax was observed 2–7 h after administration (Yu et al., 2019). Singh et al. determined that the limited ability of bergenin to be absorbed by the body may be due to its susceptibility to breakdown in the digestive system (Singh et al., 2024). The *in vivo* origin of bergenin was established by the mechanisms occurring in the human body during normal or pathological conditions. The bioavailability of bergenin was determined by administering this bioactive compound at doses of 50 mg/kg and 5 mg/kg, using the oral and intravenous routes, respectively. Following oral administration of bergenin at a dose of 12 mg/kg, the whole amount is excreted in the bile within 24 h. The majority of bergenin, approximately 97.67%, is eliminated during the first 12 h. Within 24 h, approximately 8.97% of the medication is recovered in the bile. Within the first 24 h, 95.69% of the urine included the drug, and only 22.34% of the drug was retrieved within that same period. A 100% recovery of bergenin and its glucuronide metabolite was identified, with 52.51% recovered overall. Specifically, 32.20% was found in urine within 48 h and 20.31% in bile within 24 h (Madaan et al., 2022). The effectiveness of pharmacokinetic tests was determined by measuring the concentration of bergenin in the plasma of rats following intravenous administration at dosages of 30.0, 15.0, and 7.5 mg/kg, using water as the solvent. The doses administered are not correlated with the half-lives for elimination and distribution. The plasma concentration-time graph evaluated a biphasic event characterized by rapid distribution and a prolonged elimination phase. The kinetics of this event were found to follow first-order kinetics. The Vc (central volume of distribution) was 0.67 L/kg, but the Vp (peripheral distribution) was 11.35 L/kg. The presence of bergenin in rats was found to be widely distributed. The T1/2β of bergenin in rats was 4.13 h, indicating a moderate elimination rate (Mehta et al., 2022).

## 4 Anticancer potential of bergenin

Plants produce bergenin and other natural compounds to adapt to abiotic and biotic challenges. Natural products are a compelling alternative to synthetic medicines because of their proven safety record and ability to target cancer-related characteristics such as inflammation and angiogenesis (Valakatte et al., 2024). These compounds also serve to attract animals and provide protection against ultraviolet radiation. Bergenin has garnered growing interest in recent years because of its occurrence in food and medicinal plants, such as the Amazonian herb “uchi” (Endopleura uchi) (Dhalwal et al., 2008). The fruit of this plant serves both as a food and a medicinal product. Additionally, the oil extracted from its seeds can be used in cooking and treating sinusitis in children and constipation in adults. The seeds of this plant are utilized in the production of handicrafts, for smoking foods, and as amulets (Okoro et al., 2021). Research on uchi fruit pulp has shown that it contains diverse nutrients, including fatty acids, fiber, steroids, mineral salts, and vitamins C and E. Furthermore, research indicates that bergenin possesses several qualities, such as antimalarial, antidiabetic, antioxidant, antiviral, and anti-inflammatory activity (Zhang et al., 2013). The impact of bergenin on oxidative stress and apoptosis were assessed in H9c2 cells (embryonic rat cardiomyocytes), produced by oxygen-glucose deprivation as a model of acute myocardial infarction. Bergenin exhibited a dose-dependent reduction in the impact of oxygen-glucose deprivation by decreasing ROS and MDA production while simultaneously boosting the amount of superoxide dismutase (SOD). Western blot and flow cytometry and analysis demonstrated that bergenin reduced cell death generated by oxygen-glucose deprivation by activating the SIRT1/FOXO3a/MnSOD pathway (Zhang and Chai, 2022). Idiopathic pulmonary fibrosis (IPF) can significantly impair lung function, resulting in life-threatening outcomes, and there is currently a shortage of targeted treatment medications.

Bergenin is an isocoumarin molecule with several biological properties, notably antioxidant action. Bergenin mitigated the development of lung fibrosis caused by bleomycin by alleviating oxidative stress, decreasing the accumulation of the extracellular matrix (ECM), and suppressing the generation of myofibroblasts. Additionally, bergenin stimulated phosphorylation and expression of p62 and activate Nrf2. It was shown that Nrf2 is necessary for the upregulation of p62 induced by bergenin, and the reduction of p62 through knockdown resulted in a decrease in bergenin-induced Nrf2 activity. Significantly, the antioxidant activity of bergenin and its ability to prevent TGF-β-induced ECM deposition and myofibroblast differentiation gets nullified by knocking down Nrf2 or p62. Consequently, p62 and Nrf2 established a regulatory loop for treating pulmonary fibrosis with bergenin as a critical target (Zeng et al., 2022). Bergenin has shown good anticancer potential against numerous carcinomas, including cervical cancer, colorectal cancer, bladder cancer, Oral squamous cell cancer (OSCC), non-small cell lung cancer (NSCLC), osteosarcoma, and prostate cancer (Table 3).

**TABLE 3:** Anticancer potential of bergenin against numerous human carcinomas.

Cancer	Cells	Effects of bergenin treatment	Concentration	Reference
Cervical cancer	HeLa cells	Growth inhibitory effects Reduced cell viability Increased Bax and ROS level Reduced Bcl-2 DNA Damage	IC50 = 20 μmol/mL	Gu et al. (2016)
Cervical cancer	SiHa Cells	Triggered autophagy and apoptosis Growth arrest at the G2/M phase Activation of mitophagy	IC_50_ = 15.538 μg/mL	Sultana et al. (2022)
Cervical cancer	HeLa cells	Hinderd cellular growth and STAT3 phosphorylation Triggered G0/G1 cell cycle arrest Increased Bax level Reduced Bcl-2 levels	IC_50_ = 15 µM	Shi et al. (2019)
Ovarian granulosa cells	Ovarian granulosa cells	Control oxidative stress by activating the Nrf2 pathway Apoptosis induction	Dosage = 50, 100 mg/kg	Zhu et al. (2024)
Breast cancer	MDA-MB-231 cells	Inhibited the cytoprotective unfolded protein response Boosted PERK-ATF4-CHOP pathway Induced apoptosis Blocked IRE1-Xbp1 and ATF6 pathways	Dosage = 50–125 μg/mL	Qadri et al. (2023)
Prostate cancer	PC3, LNCaP and DU145 cells	Promoted apoptotic cell death Increased Sub G1 population via Increasing mitochondrial outer membrane permeability Enhanced ROS levels Activation of the β-TrCP-GSK-3β axis Inactivation of the NRF2-antioxidant response pathway Activity of GSK-3β	IC_50_ of PC3 and LNCaP cells = 87.0 μM and 50.0 μM and IC_50_ of NKE and WI38 cells = 852.6 μM and 810.2 μM for	Ghosh et al. (2021)
Liver cancer	HepG2 cells	Growth suppressor and inhibitor of Akt/Bcl-2 G2/M phase arrest Triggered apoptosis Mitochondrial mediated apoptosis	IC_50_ = 4.23 μM	Liang et al. (2017)
Liver cancer	HepG2 cells	Protection against oxidative stress induced by sodium selenite Decreasing lipid peroxidation Restored the equilibrium between oxidants and antioxidants	Dosage = 10, 50, and 250 mg/kg/day	Sriset et al. (2021a)
Bladder cancer	bladder cancer cells	Inhibited metastasis via activating the PPARγ/PTEN/AKT signaling pathway Increase in G1 phase arrest Reduced expression of Ki67, cyclin D1, and cyclin B1, Bcl-2 Elevated levels of Bax and cleaved caspase-3 proteins	Dosage = 12.5 and 25 μM	Liu et al. (2021)
Urinary bladder cancer	T24 cells	Apoptosis induction and cell death Modified anti- and pro-apoptotic proteins Activation of caspase-3 Mitochondrial malfunction	IC_50_ = 14.36 μM	Zhang et al. (2018)
Non-small cell lung cancer	NSCLC cells	Hinders survivin phosphorylation Controlling the Akt/Wee1/CDK1 signaling pathway Activation of cleaved-PARP and caspase 3 Apoptosis induction	IC_50_ = 20 μM for 24 h and IC_50_ = 4 0 μM	Li et al. (2022)
Non-small cell lung cancer	NSCLC cells	Facilitating the ubiquitination process and subsequent survivin degradation Suppressed the AKT/Wee1/CDK1 signaling pathway Reduced phosphorylation of survivin	IC_50_ = 25 μM	Li et al. (2023a)
Oral squamous cell cancer	OSCC cells	Suppressed glycolysis, hence overcoming radioresistance Lowered expression of HK2 Decelerated metabolic process of glycolysis Induced intrinsic apoptosis	Dosage = 25 μM and 30 μM	Li et al. (2023b)
Colorectal adenocarcinoma	HCT116 cancer cells	Growth inhibition via blocking PI3K/AKT/mTOR pathway G1 growth phase arrest DNA damage Increased ROS levels	Doses = 3, 10, 30 μM	Gao et al. (2017)
Colorectal cancer	CRC cells	Decreasing their viability and colony Growth suppression via promoting the degradation of Mcl-1 Apoptosis induction by FBW7-mediated Mcl-1 ubiquitination	IC_50_ = 30 μM	Gan et al. (2023)
Osteosarcoma	MG63 cells	Suppressed the growth of MG63 cells Triggered programmed cell death Increased levels of cleaved-PARP, cleaved-caspase 3, TUNEL-positive cells, p53, p21, LC3II and Annexin V-positive cells Decrease in Bcl-2 and cdk2 deactivating AKT promoting the production of autophagosomes	Dosage = 75, 150, and 300 μM	Park et al. (2021)

### 4.1 Bergenin efficacy against gynecologic cancer

Bergenin exhibited a dose-dependent effect on the viability of HeLa cervical cancer cells, causing a reduction in cell viability. However, the therapeutic impact of bergenin on normal cervical cells was observed to be relatively diminished. Moreover, the primary mechanism by which bergenin exhibits its anticancer properties is the induction of apoptosis in HeLa cervical cancer cells. Significantly, bergenin also increased the expression of Bax and reduced the expression of Bcl-2. The impact of bergenin on the distribution of cell cycle phases in HeLa cells was also examined, revealing that bergenin can trigger G0/G1 cell cycle arrest. In addition, bergenin can hinder the movement of HeLa cancer cells and the process of phosphorylation of STAT3. Bergenin shows potential as a viable option for treating cervical cancer (Shi et al., 2019). The water-based extract of *E. agallocha* effectively reduced the growth of SiHa cervical cancer cells by triggering autophagy and apoptosis. The simultaneous activation of mitophagy and cell growth arrest at the G2/M phase accompanied this process. These findings suggest that Bergenin, the primary compound in the plant extract, is responsible for its anti-cancer properties (Sultana et al., 2022). Mcl-1, a protein associated with myeloid leukemia 1, is commonly expressed at high levels in several types of cancer and has shown potential as a target for pharmacological therapy. Additional research data reveals a new understanding of how bergenin inhibits the growth of blood vessels in cervical carcinoma cells by affecting various proteins involved in blood vessel formation, such as Galectin-3 and MMP-9. These findings support the potential use of bergenin as a treatment for cervical cancer.

*Bergenia ciliata* (haw.) Sternb is a widely recognized medicinal plant that has long been utilized for its therapeutic effects in treating various conditions, including diabetes, microbiological infections, and kidney stones. This is due to its notable anti-inflammatory and anti-tussive characteristics. B. *ciliata* possesses strong anti-cancer characteristics due to its capacity to regulate the unfolded protein response (UPR) and ROS pathways. The methanolic extract of B. ciliata (BcME) was tested on MDA-MB-231 and C6-Glioma cell lines. The plant extract specifically inhibited the cytoprotective UPR by blocking the IRE1-Xbp1 and ATF6 pathways while simultaneously boosting the PERK-ATF4-CHOP pathway, which is known to shift the UPR towards apoptosis. In addition, B. ciliata substantially impacted ROS formation in cancerous cells and hindered the activity of antioxidant enzymes superoxide dismutase and catalase. The findings strongly indicate that BcME cooperates with the UPR and ROS pathways to enhance apoptosis and eradicate cancer cells. Therefore, it can potentially be a valuable source of bioactive compounds targeting cancer cells (Qadri et al., 2023). Bergenin efficiently inhibited oxidative stress and apoptosis in ovarian granulosa cells (OGCs) by regulating the Nrf2 signaling pathway. In conclusion, this study reveals that bergenin can effectively control oxidative stress by activating the Nrf2 pathway, decreasing apoptosis of OGCs, and alleviating ovarian damage in POF mice induced by triptolide. However, the authors could not confirm the role of Nrf2-mediated oxidative stress in the protective effect of bergenin against premature ovarian failure (POF) in our animal trials. To overcome this constraint, future studies can establish a method to deactivate Nrf2 in OGCs, allowing the required validation selectively (Zhu et al., 2024).

It is specifically a C-glycoside of 4-O-methylgallic acid. Reverse docking calculations were utilized to identify potential biological targets of bergenin. The reverse docking analysis indicated that bergenin can target galectin-3. Gelectin-3 is an enzyme that has significant involvement in various cellular processes such as cell-to-cell adhesion, interactions between cells and the extracellular matrix, activation of macrophages, formation of new blood vessels, metastasis, and programmed cell death (apoptosis). Because of its crucial function in cancer, Gelectin-3 is a widely studied target for developing drugs that help combat cancer. The interaction between bergenin and galectin-3 was examined using traditional forward docking calculations. The stability of the galectin-3: bergenin complex was investigated using classical molecular dynamics (MD) simulations. Computations of docking and MD analysis revealed that bergenin can firmly attach to the carbohydrate recognition domain (CRD) of galectin-3. The binding of bergenin to galectin-3 also involves a few robust hydrogen bonds.

Furthermore, a credible π-stacking interaction exists between the aromatic component of bergenin and the His158 residue located at the binding site. A 50-nanosecond molecular dynamics simulation was conducted for the bergenin: galectin-3 complex in a water box with periodic boundary conditions. The MD analysis demonstrated excellent stability of bergenin: galectin-3 complex and validated the accuracy of docking results. These findings supported the hypothesis that bergenin have an inhibitory impact on galectin-3. This study provided fresh insights into the anticancer properties of bergenin and highlighted its potential as a promising candidate for developing more powerful galectin-3 inhibitors. The study offered scientific evidence that supports using plants containing bergenin in cancer treatments within Eastern traditional medicine (Jayakody et al., 2018).

### 4.2 Bergenin efficacy against prostate cancer

The polyphenol-rich fraction of Bergenia ligulata (PFBL) is derived from a plant commonly utilized in Indian folk and traditional medicine due to its anti-inflammatory and antineoplastic effects. The LCMS study revealed that PFBL is composed of approximately fifteen distinct compounds. PFBL promoted apoptotic cell death in both androgen-dependent LNCaP cells androgen-refractory PC3 and DU145 cells. However, it had no impact on NKE and WI38 cells. Subsequent examination uncovered that PFBL performed its function via increasing ROS through the improved catalytic activity of Monoamine oxidase A (MAO-A). The differential inactivation of the NRF2-antioxidant response pathway by PFBL led to the death of PC3 cells compared to NKE cells. This process involved the activity of GSK-3β, which was assisted by the inhibition of AKT. PFBL effectively decreased the size of PC3-tumor xenografts in NOD-SCID mice both on its own and in combination with Paclitaxel, resulting in a synergistic effect. The tumor tissues in mice treated with PFBL exhibited an increase in the same cell death method found in isolated PC3 cells. This mechanism involved an elevation in the catalytic activity of MAO-A, the creation of ROS, and the activation of the β-TrCP-GSK-3β axis, which leads to the degradation of NRF2. The safety of PFBL in healthy mice was confirmed through tests of blood counts, liver function, and splenocyte sensitivity (Ghosh et al., 2021).

11-O-Galloyl bergenin (OGAL) was extracted from the leaves of Corylopsis coreanas and tested on human osteosarcoma cells. In this study, we discovered that OGAL effectively suppressed the growth of MG63 cells and triggered programmed cell death, as indicated by the presence of cleaved-PARP, cleaved-caspase 3, TUNEL-positive cells, and Annexin V-positive cells. An increase in p53 and p21 levels, BAX expression, and a decrease in Bcl-2 and CDK2 characterized OGAL-induced apoptosis. Furthermore, OGAL triggered autophagy by deactivating AKT, increasing the expression of LC3II, and promoting the production of autophagosomes in MG63 cells. OGAL-induced autophagy was observed to be associated with elevated p38 phosphorylation, but the activities of JNK and ERK1/2 remained unaltered when investigating the MAPK signaling pathway. In addition, experiments on wound healing and Boyden chamber assays demonstrated that OGAL inhibited the migration and invasion of MG63 cells. The study showed that OGAL exhibits anticancer effects by enhancing apoptosis and autophagy through upregulation of p53, AKT, and p38 signaling. These findings suggested that OGAL could be a promising therapeutic approach for treating osteosarcoma (Park et al., 2021).

### 4.3 Bergenin efficacy against neuronal cancer cells

Inflammation is a defensive biological response to infection and has adverse effects. Despite the numerous pharmacological qualities of bergenin, a derivative of 4-O-methyl gallic acid, there is currently no study that has shown the effectiveness of bergenin in reducing inflammatory mediator levels, including prostaglandin E2 (PGE2) and histamine. Prior administration of bergenin significantly decreased edema caused by carrageenan, compound 48/80, histamine, and PGE2. Furthermore, the safety of bergenin administration is reassuring. The administration of bergenin (2,000 mg/kg) did not result in any observable indicators of toxicity in the screening of vital signs, animal weight, water and food consumption, the weight of significant organs, histological examination, and hematological and biochemical parameters. These findings demonstrate that the bergenin derived from *P. dubium Taub* possesses anti-inflammatory properties against various inflammation models. It effectively decreases neutrophil migration and mitigates the deleterious effects of lipid peroxidation and oxidative stress (Oliveira et al., 2019). Oxytosis refers to an imbalance between the production and expression of reactive oxygen species and the capacity of a biological system to repair the associated damage. Bergenin provided cellular protection against glutamate-induced toxicity at a concentration of 10 μM, and its effectiveness increased in a dose-dependent manner until reaching maximum protection at 30 μM. As shown by a cell viability experiment, prior administration of bergenin significantly inhibited the increase of glutamate-induced reactive oxygen species. It protected HT22 (murine neuronal cell) cells from glutamate-induced cell death. Protection is diminished by bergenin due to the restoration of glutathione levels. In addition to increasing glutathione activity, the antioxidant’s capacity to eliminate ROS also triggers the activation of the Peroxisome Proliferator-Activated Receptor, peroxisome proliferator-activated receptor gamm (PPARγ), which regulates many proinflammatory cytokines (Shukry et al., 2019).

### 4.4 Bergenin efficacy against hepatocellular malignancy

Hepatic ischemia-reperfusion (IR) hinders the advancement of liver transplantation technology. Bergenin suppressed the release of ROS, decreased the expression of inflammatory markers, and hindered apoptosis and autophagy. In the Bergenin pre-treatment groups, the expression of PPAR-γ-related genes was enhanced. In contrast, the phosphorylation of P38 MAPK, NF-κB p65, and JAK2/STAT1-related proteins was reduced depending on the dosage. Bergenin provided hepatic protection in this model of hepatic ischemia-reperfusion injury by reducing ROS, modulating the release of inflammatory molecules, and regulating the expression of genes linked to apoptosis and autophagy through the PPAR-γ pathway (Xiang et al., 2020). Bergenin’s phenolic hydroxyl groups were shielded using benzyl bromide to form benzyl-protected bergenin. The dibenzyl bergenin compound was subjected to acid treatment in the presence of EDC·HCl and DMAP in CH_2_Cl_2_. Subsequently, the resulting product was hydrogenated using Pd/C catalysts, resulting in bergenin ester derivatives. Multiple bergenin derivatives, with triple substitutions at positions 3a, 4a, 5a, 6a, and 7a, and double substitutions at positions 8b and 9b, showed more significant cytotoxicity than bergenin. The study demonstrated that the cytotoxic activity of bergenin esters is significantly influenced by the size of substituents and the lipophilicity of the compounds (De-Biao et al., 2014).

Bergenin is a compound derived from gallic acid known for its antioxidant and hepatoprotective properties. Sodium selenite caused damage to HepG2 cells and *in vivo* models by disrupting the equilibrium between oxidants and antioxidants. Bergenin had a defensive impact against oxidative stress induced by sodium selenite in HepG2 cells. In addition, bergenin mitigated the liver damage caused by sodium selenite by reducing alkaline phosphatase, plasma aspartate aminotransferase, and alanine aminotransferase. It also produced ROS, decreasing lipid peroxidation in both plasma and liver tissues. This resulted in a reduction in nuclear pyknosis and necrotic areas in the liver. Bergenin also normalized the levels and activities of liver antioxidant enzymes (superoxide dismutase, catalase, and glutathione peroxidase) and restored the balance of hepatic glutathione. Ultimately, bergenin restored the expression of cytochrome P450 2E1 mRNA to its normal levels. Overall, bergenin effectively mitigated liver damage caused by reduced sodium selenite by restoring the equilibrium between oxidants and antioxidants and decreasing lipid peroxidation through various mechanisms (Sriset et al., 2021a).

The hepatoprotective effects of bergenin were investigated against oxidative stress induced by ethanol and tert-butyl hydroperoxide (TBHP) in HepG2 cells. HepG2 cells were simultaneously exposed to ethanol or TBHP (100 µM) and either bergenin or gallic acid for 24 h. Both ethanol and TBHP caused damage to HepG2 cells, resulting in the loss of cell nuclei as observed through cell morphology. Exposure of HepG2 cells to either ethanol or TBHP resulted in a decrease in SOD and catalase (CAT) activities and a depletion of total glutathione (GSH) content in comparison to the control. Bergenin and gallic acid strengthened the physical structure of cells, increased cell survival, reduced lactate dehydrogenase (LDH) toxicity, restored aspartate aminotransferase (AST), alanine transaminase (ALT), and malondialdehyde (MDA) levels, stimulated SOD and CAT activities, and improved the overall GSH content of HepG2 cells treated with ethanol and TBHP. Bergenin demonstrated hepatoprotective action by restoring the oxidant-antioxidant system, making it a promising candidate for hepatoprotective treatment (Sriset et al., 2021b). Fourteen bergenin/cinnamic acid hybrids were synthesized, described, and assessed for their antitumor activity in laboratory settings and living organisms. The researchers synthesized novel bergenin and cinnamic acid hybrids and assessed their antitumor effectiveness through *in vitro* and *in vivo* experiments. Chemical 5c demonstrated significant efficacy in stopping the growth of HepG2 cells in the G2/M phase and triggering apoptosis through the mitochondria. This chemical is a promising inhibitor of Akt/Bcl-2 and should be further investigated in preclinical research (Liang et al., 2017).

### 4.5 Bergenin efficacy against bladder cancer

The administration of Bergenin resulted in a substantial reduction in cell viability and an increase in G1 phase arrest in bladder cancer cells. This was accompanied by a decrease in the expression of Ki67, cyclin D1, and cyclin B1. Bergenin caused apoptosis in bladder cancer cells, as demonstrated by elevated levels of Bax and cleaved caspase-3 proteins and reduced levels of Bcl-2 in cells treated with bergenin. The mechanism investigations demonstrated that bergenin administration effectively stimulated the PPARγ/PTEN/AKT signal pathway, as evidenced by the upregulation of nuclear PPARγ and phosphatase and tensin homolog (PTEN) expression and the downregulation of AKT expression. Furthermore, the treatment of a PPARγ inhibitor reversed the effects of bergenin on the activity of bladder cancer cells, namely, in terms of proliferation, apoptosis, invasion, and migration. The study showed that bergenin can hinder the advancement of bladder cancer by activating the PPARγ/PTEN/AKT signaling pathway. This suggests that bergenin can be used as a therapeutic medication for treating bladder cancer (Liu et al., 2021).

The dried leaves of *Bergenia purpurascens* were used to produce ethanolic extracts. These extracts were then analyzed, discovering and identifying a new aromatic glycoside called 1-O-β-D-glucopyranosyl-2-methoxy-3-hydroxyl-phenylethene.Additionally, 19 additional compounds (2–20) that were already recognized were also found in the extracts. Compound 1’s structure was elucidated through a comprehensive examination utilizing various analytical techniques (D and 2D NMR). Chemical 1 was assessed for its *in vitro* growth inhibitory efficacies against selected human cancer cells. Compound 1 exhibited the highest potency level, as indicated by its IC50 values of 14.36 ± 1.04 μM against T24 cells. Subsequent bioactivity research revealed that compound 1 triggered apoptosis in T24 cells (urinary bladder cancer) by modifying anti- and pro-apoptotic proteins, resulting in mitochondrial malfunction and the activation of caspase-3, ultimately leading to cell demise. This study showed that compound 1 can be utilized as a candidate for antitumor chemotherapy (Zhang et al., 2018).

### 4.6 Bergenin efficacy against lung carcinoma

A novel bergenin derivative, 4-O-p-hydroxybenzoylbergenin (compound 1), was extracted from whole plants of *Ardisia maclurei Merr.,* in addition to three previously identified compounds: wogonin (compound 2), wogonoside (compound 3), and α-spinasterol (compound 4). Compound 1 demonstrated inhibitory effects on A549 cells, with an IC50 value of 20 μmol/mL, as reported by Gu et al., in 2016. Bergenin efficiently inhibited the proliferation of colorectal adenocarcinoma cells via stimulating ROS within the cells, causing DNA damage and cell growth arrest during the G1 phase. This action is achieved by blocking the PI3K/AKT/mTOR pathway (Gao et al., 2017). Survivin is highly expressed in various types of human cancers and is associated with an adverse prognosis. Bergenin inhibited the growth and development of cell colonies in A549, H1299, and H460 cells and decreased the amount of survivin protein in a manner that depends on the dosage. The mechanistic study demonstrated that bergenin hindered the phosphorylation process of survivin by controlling the Akt/Wee1/CDK1 signaling pathway. As a result, it strengthened the connection between survivin and E3 ligase Fbxl7. Furthermore, bergenin amplified the activation of cleaved-PARP and caspase 3, hence facilitated the process of apoptosis in NSCLC cells. In the *in vivo* investigation, it was observed that the tumor volume, weight, and expression of Ki67 and survivin were significantly reduced in the group treated with bergenin. These data demonstrated that bergenin exhibits a notable antitumor effect and is promising as a treatment drug for NSCLC (Li et al., 2022). Hexokinase 2 (HK2), the primary enzyme that controls the speed of glycolysis, is closely linked to the growth and progression of cancer. There is a pressing demand for therapeutic medications that specifically target HK2. Bergenin demonstrated a suppressive impact on the development of cancer cells, decelerated the metabolic process of glycolysis, and induced intrinsic apoptosis in OSCC via lowering the expression of HK2. Bergenin effectively restored the efficacy of radiation therapy in the radio resistant OSCC cells. A comprehensive analysis revealed that bergenin augmented PTEN protein level by promoting the association between PTEN and ubiquitin-specific protease 13 (USP13), thereby boosting the stability of PTEN. Consequently, this pathway ultimately impeded the phosphorylation of protein kinase B (AKT) and inhibited the production of hexokinase 2 (HK2). Bergenin has been identified as a novel therapeutic medication that can efficiently suppress glycolysis, hence overcoming radio resistance in OSCC (Li et al., 2023a).

Chemoresistance significantly contributes to treatment failure and tumor recurrence in persons diagnosed with NSCLC. Survivin, a protein associated with cancer, is often found at high levels in human tumors and is associated with a poor prognosis. Survivin overexpression is essential for maintaining many malignant traits of NSCLC cells. Bergenin suppressed the AKT/Wee1/CDK1 signaling pathway, resulting in a reduction in the phosphorylation of survivin. Consequently, this led to an enhanced association between survivin and E3 ligase Fbxl7, facilitating the ubiquitination process and subsequent survivin degradation. Furthermore, bergenin augmented the resilience of NSCLC cells that had been rendered susceptible to pemetrexed treatment once more. Bergenin exerts a potent anticancer effect on NSCLC by selectively targeting survivin. Therefore, it has excellent potential as a therapeutic medication for treating NSCLC (Li et al., 2023b).

### 4.7 Bergenin efficacy against colorectal cancer

**FIGURE 3:**
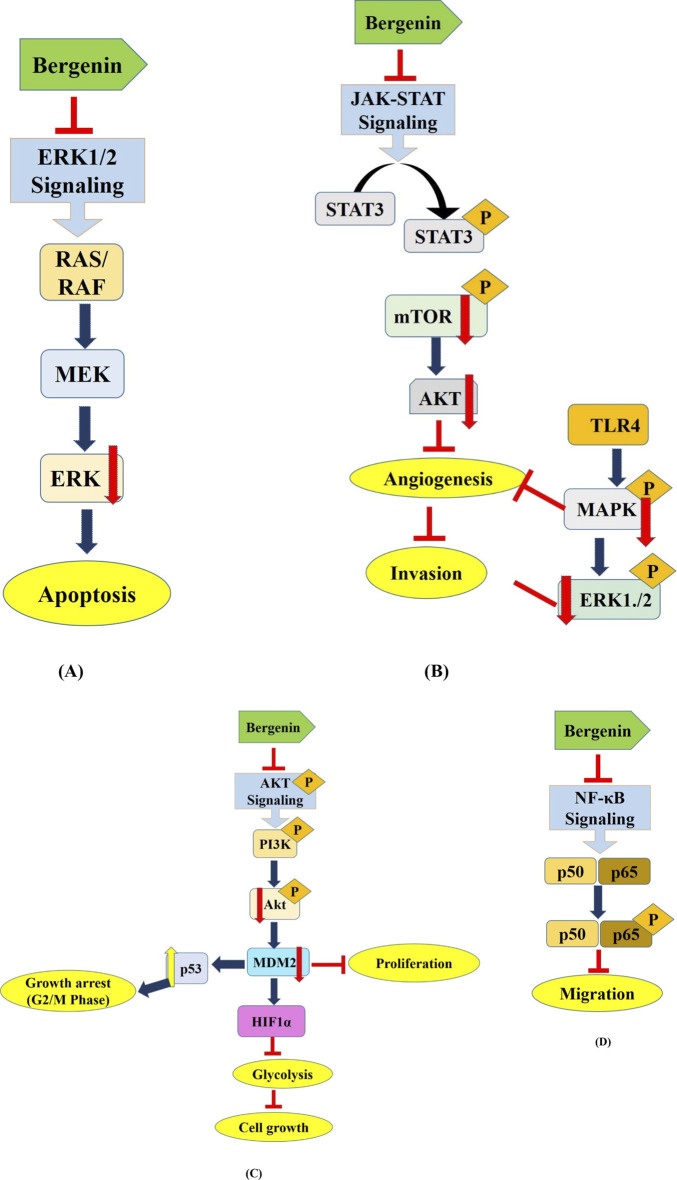
Deregulated cell signaling pathways targeted by Bergenin. This figure hypothesizes the mechanism of action associated with the anticancer potential of bergenin against several types of human cancers via targeting several deregulated components of cell signaling pathways including **(A)** ERK1/2 (Park et al., 2021), **(B)** JAK/STAT (Shi et al., 2019), **(C)** STAT3/Akt (Shi et al., 2019; Liu et al., 2021), **(D)** NF-kB (Xiang et al., 2020) and associated with the progression of human cancers. Bergenin has been reported to modulate the oncogene and tumor suppressor gene expression to inhibit the carcinogenesis. Modulation of these dysregulated components leads to several mechanisms that inhibit the growth of cancer cells such as apoptosis induction, growth arrest at G0/S/M phases, angiogenesis and glycolysis inhibition. PTEN, Phosphatase and TENsin homolog; AKT, AKT serine-threonine protein kinase family; mTOR, mammalian target of rapamycin; MAPK, mitogen-activated protein kinase; STAT-3, Signal transducer and activator of transcription 3.

HCT116 cancer cells were exposed to different doses of bergenin during 24 and 48 h. Bergenin exerted a pronounced inhibitory effect on the viability of HCT116 cells. In addition, bergenin caused cells to gather in the G1 phase and led to DNA damage in HCT116 cells. Additionally, it resulted in a significant buildup of intracellular ROS, which is known to cause DNA strand breakage in HCT116 cells. Remarkably, bergenin suppressed the PI3K/AKT/mTOR pathway. In conclusion, Bergenin efficiently inhibited the growth of colorectal adenocarcinoma by causing the production of ROS inside cells, damaging DNA, and resulting in G1 growth phase arrest via blocking the PI3K/AKT/mTOR pathway (Gao et al., 2017). Bergenin exhibited a dose-dependent effect on colorectal cancer (CRC) cells by drastically decreasing their viability and colony formation and promoting apoptosis. Bergenin triggered programmed cell death in colorectal cancer cells using FBW7-mediated Mcl-1 ubiquitination. *In vivo* experiments demonstrated that bergenin effectively decreased the weight and size of tumors in mice while not causing any harm to essential organs. In summary, bergenin suppressed the growth of CRC cells by promoting the degradation of Mcl-1. This indicates that interfering with the process of Mcl-1 ubiquitination could serve as a viable approach for anticancer therapy, as proposed by Gan et al., in 2023. Altogether, this review provides a fresh insights into the anticancer properties of bergenin and highlighted its potential as a promising candidate for using plants containing bergenin in cancer treatments. Figure 3 describes the mechanism of action associated with the anticancer potential of bergenin against several types of human cancers.

## 5 Anticancer potential of bergenin nanoformulations

The burgeoning field of biological nanoparticle synthesis is gaining traction due to its myriad pharmaceutical and therapeutic applications. Bergenin has demonstrated significant efficacy against a wide range of chronic ailments. However, bergenin ’s pharmacological significance and therapeutic effectiveness are restricted due to its inadequate oral absorption, low solubility in water, and limited capacity to pass across membranes. To overcome these obstacles in the pharmaceutical field, it is necessary to devise novel formulation procedures. Lately, the scientific community has been primarily concerned with developing nano-based carrier systems. The innovative approaches encompass phospholipid complex, herbal gel, extended-release core tablets, phospholipid complex solid dispersion, prodrug, polyherbal ointment, nanoparticles, and poly lactic acid polymers. Given the constraints, scientists have developed multiple drug delivery systems to improve its effectiveness in treatment. Despite the extensive utilization of bergenin, its limited oral bioavailability remains a barrier to its continued employment. Bergenin, classified as a class IV chemical, exhibits insufficient solubility and permeability to achieve total absorption (Bajracharya, G. B., 2015). Various strategies have been employed to enhance the bioavailability of the substance, including structural modifications, prodrugs, and the development of new dosage forms such as β-cyclodextrin inclusion complexes and phospholipid complexes (Gao et al., 2018; Stella, V. J., & Rajewski, 2020).

Earlier studies have presented that reduced particle size of bergenin employing nanosuspensions can promote its solubility (Rao et al., 2018). Applying BgAgNps (Bergenia ligulata silver nanoparticles) resulted in significant cytotoxic effects on MCF-7 cells, decreasing cell viability, migratory capacity, and the loss of typical morphological features. BgAgNps demonstrated a notable increase in programmed cell death in MCF-7 breast cancer cells, potentially triggering ROS production through oxidative stress and causing a decline in mitochondrial membrane potential (MMP). In addition, investigations based on molecular mechanisms have shown that BgAgNps significantly increased the levels of p53 and its downstream targets, such as Bax and cleaved caspase 3, in MCF-7 cells. It is worth mentioning that BgAgNps did not have a significant cytotoxic impact on p53-deficient cancer cells, specifically MDA-MB-231 and SW-620 (Faheem et al., 2022).

A nanostructured lipid carrier (NLC) containing bergenin was formulated to enhance the effectiveness of oral therapy. The results of the solid-state characterization demonstrated that bergenin was fully enclosed within the NLC matrix. The improved bergenin NLC demonstrated a considerably longer release duration than pure bergenin. The Korsmeyer-Peppas model was determined to be the most suitable kinetic model. Additionally, it was shown that the formulation had a 3.2 times higher rate of movement in the intestines than pure bergenin, with improved penetration across the membrane studied. The relative bioavailability findings of bergenin NLC show a markedly superior bioavailability compared to pure bergenin. The improved formulation achieved the most significant reduction in swelling when compared to pure bergenin and the conventional medication indomethacin. The formulation had a prolonged effect, lasting for 12 h. These results suggested that including bergenin in the NLC matrix might enhance the effectiveness of treatments (Zafar et al., 2024).

In a separate study, Guan et al. investigated the mechanism by which the bergenin–phospholipid combination enhances the oral absorption of bergenin. Diverse models utilizing the everted rat gut sac (*ex vivo*) and Caco-2 cells (*in vitro*) were utilized. The results from the *ex vivo* model of everted rat gut sac indicated that the small intestine is more favorable for the absorption of this bioactive substance compared to the colon. Moreover, the incorporation of chitosan facilitated the opening of tight junctions in intestinal epithelial cells, hence significantly improved the transport of bergenin via the paracellular pathway (Gao et al., 2018). The field of synthesizing metal and semiconductor nanoparticles is growing as researchers explore their possible applications in advancing new technologies. This study presents a new method for synthesizing zinc oxide nanoparticles using the rhizome extract of the plant *B. ciliata*. The study conducted by Dulta et al. (2021) demonstrated that ZnO nanoparticles had a notable and specific toxicity towards the HeLa and HT-29 cell lines, which are associated with human cervical and colon cancer, respectively. Additional examination revealed that bergenin is a powerful anti-angiogenic substance, as it decreased the levels of essential angiogenic proteins, such as Galectin 3 and MMP-9, in cervical cancer cells at the cellular level, as demonstrated by a western blotting assay. These data collectively offer a new understanding of how Bergenin inhibited the growth of blood vessels in cervical carcinoma cells. This is achieved by affecting the activity of various proteins involved in blood vessel formation, such as Galectin-3 and MMP-9. These findings supported the potential use of Bergenin as a treatment for cervical cancer (Singh et al., 2024). The efficacy of this phytochemical is involved in various mechanisms, including inhibiting lipid peroxidation, scavenging free radicals, initiating apoptosis and arresting growth at G0/G1 phase, inhibiting STAT3 proteins phosphorylation, inducing the production of TNF-α, NO, IFN-γ, IL-17, IL-12, and inhibiting the α-glucosidase enzyme.

## 6 Conclusion and future perspective

Plant-derived secondary metabolites have garnered significant attention as bioactive compounds over the years. This review aimed to outline the anticancer effects of bergenin on several cancer types by focusing on apoptosis, angiogenesis, and the suppression of dysregulated cell signaling pathways, including ERK1/2, MAP kinase, and SAPK/JNK. The anti-cancer action of bergenin has also been documented through the modulation of many angiogenic proteins, including STAT-3 and MMP-9. Furthermore, nanoformulations of bergenin have inhibited its depletion in healthy tissues. However, future research is also required to explore the synergistic effects of bergenin with additional natural compounds or established chemotherapeutic agents. Consequently, researchers must concentrate not only on the efficacy of bergenin, which is of significant interest, but also on drug delivery systems capable of addressing pharmacokinetic challenges, as well as investigating derivatives with enhanced biological activity and bioavailability.

## Glossary

**ROS**: Reactive oxygen species
**ECM**: Extracellular matrix
**HIA**: Human intestinal absorption
**CaCo-2**: Human colon adenocarcinoma cell line
**PPB**: Plasma protein binding
BBB: Blood-brain barrier
**CNS**: Central nervous system
**IPF**: Idiopathic pulmonary fibrosis
**OSCC**: Oral squamous cell cancer
**NSCLC**: Non-small cell lung cancer
**UPR**: Unfolded protein response
**PPARγ**: Peroxisome proliferator-activated receptor gamma
**MD**: Molecular dynamics
**CRD**: Carbohydrate recognition domain
**POF**: Premature ovarian failure
**OGCs**: Ovarian granulosa cells
**PFBL**: Polyphenol-rich fraction of Bergenia ligulata
**MAO-A**: Monoamine oxidase A
**OGAL**: O-Galloyl bergenin
**PGE2**: Prostaglandin E2
**IR**: Ischemia-reperfusion
**TBHP**: Tert-butyl hydroperoxide
**HepG2**: Human hepatoma cells
**SOD**: Superoxide dismutase
**CAT**: Catalase
**GSH**: Glutathione
**LDH**: Lactate dehydrogenase
**AST**: Aspartate aminotransferase
**ALT**: Alanine transaminase
**MDA**: Malondialdehyde
**PTEN**: Phosphatase and tensin homolog is a phosphatase in humans
**USP13**: Ubiquitin-specific protease 13
**AKT**: Protein kinase B
**HK2**: Hexokinase 2
**CRC**: Colorectal cancer
